# COVID-19 Outbreak at a Geriatric Rehabilitation Facility: The Silent Threat of Asymptomatic Patients with High Viral Loads

**DOI:** 10.3390/geriatrics6040095

**Published:** 2021-09-30

**Authors:** Pauline Putallaz, Laurence Senn, Wanda Bosshard, Christophe J. Büla

**Affiliations:** 1 Service of Geriatric Medicine and Geriatric Rehabilitation, Lausanne University Hospital and University of Lausanne, 1011 Lausanne, Switzerland; Wanda.Bosshard@chuv.ch (W.B.); christophe.bula@chuv.ch (C.J.B.); 2 Service of Hospital Preventive Medicine, Lausanne University Hospital and University of Lausanne, 1011 Lausanne, Switzerland; Laurence.Senn@chuv.ch

**Keywords:** SARS-CoV-2, COVID-19, older persons, nosocomial infections, rehabilitation facility, viral load

## Abstract

Data about outbreaks of nosocomial COVID-19 disease within geriatric rehabilitation facilities are scarce. In this retrospective case series analysis, we describe such an outbreak, determine the proportion of a-/presymptomatic patients, the median time before symptom onset among presymptomatic patients and investigate whether the viral load differs between patients with and without symptoms. Typical and atypical symptoms were retrieved from the electronic medical records of patients who tested positive for COVID-19 disease during their stay at a 95-bed geriatric rehabilitation facility. The viral load at the time of diagnosis was estimated on cycle threshold values of the rRT-PCR test. Overall, 34 patients (median age, 87 years; range, 66–98; 67% female) tested positive for SARS-CoV-2. During the same period, 19 health care workers were also diagnosed with COVID-19. Among the 27 patients who provided consent, 20 (74%) were symptomatic at the time of testing. Among the remaining seven patients, six developed symptoms after a median of 2 days. A viral load > 10^6^ copies/mL was observed in 20 out of the 27 patients, including five out of the seven initially asymptomatic patients. The rapid transmission of SARS-CoV-2 and the prevalence of initially asymptomatic patients with high viral loads support an extended screening strategy at such facilities.

## 1. Introduction

The main route of transmission of the severe acute respiratory syndrome coronavirus (SARS-CoV-2) is respiratory, mainly by droplets, and possibly by aerosols [1,2]. SARS-CoV-2 transmission occurs from 1–5 days before up to 14 days after symptom onset, culminating in the first few days after disease onset [3,4,5].

Symptomatic patients are likely to be identified early, even though atypical symptoms such as chills, malaise, confusion, rhinorrhea, nausea or diarrhea, falls or simple changes in general condition are more frequent in older persons [6,7].

Asymptomatic patients pose a major concern as they may also transmit the infection [8,9]. For instance, several outbreaks on ships showed that asymptomatic infection rates in younger population ranges from 47.8% up to 81.3% [9]. Fewer data are available about asymptomatic infection rates among older patients. In particular, data are lacking from the rehabilitation setting where patients are encouraged to ambulate and receive care that requires close contact with healthcare workers (HCWs). Even though a systematic review found that the secondary attack rate from the symptomatic index cases contact was greater than from asymptomatic cases in home and community settings [10], transmission by asymptomatic persons remains of highest concern within geriatric facilities [3,11,12].

An additional concern relates to the paucity of data on the proportion of initially asymptomatic older persons who subsequently develop symptoms. The King Country cohort [13] and the results of Arons et al. [3] suggest that few elderly patients remain completely asymptomatic during the follow-up.

Finally, data regarding the correlation between the viral load and symptoms are conflicting. Some studies report similar viral loads in symptomatic and a-/paucisymptomatic patients [14], whereas other do not [15]. Moreover, very few data in an older population are available.

An outbreak of SARS-CoV-2 at a geriatric rehabilitation facility provided an opportunity to address some of these issues. Specifically, the aims of this study were fourfold. First, to describe this outbreak; second, to determine the proportion of asymptomatic and presymptomatic patients at the time of diagnosis; third, to determine the median time before symptom onset among presymptomatic patients; and finally, to investigate whether the viral load differed according to clinical presentation.

## 2. Materials and Methods

### 2.1. Setting

The Geriatric Rehabilitation Center is a 95-bed rehabilitation unit. The facility has six hospitalization floors (floor ##3–8), each with 14–17 beds in single or double bedrooms. Each year, this inpatient rehabilitation facility admits about 1400 patients (mean age, 86 years; 66% women) who are transferred mostly after an acute hospital stay (~90%) in orthopedic, internal medicine, and surgical wards. These patients are cared for by a multidisciplinary team of HCWs (physicians, nurses, occupational therapists, physical therapists, dieticians and psychologists). About 75% of the admitted patients achieve their objectives of restoring their mobility and functional independence to return home. The other patients are discharged to long-term care (~10–12%), transferred to acute care (~10–12%) or die (~3–5%).

### 2.2. Epidemic Context

On 25 February 2020, the first case of COVID-19 was diagnosed in Switzerland, and on 11 March 2020, the threshold of 1000 detected cases was reached.

Early in March 2020, institutional policy guidelines were issued:

- Reinforced information on hand hygiene and physical distancing;
- Suppression of dining room meals in the common area and limitation of the patients’ ambulation within the facility starting on 9 March;
- Withholding of visits and common activities starting on 13 March;
- Mandatory wearing of face masks for all HCWs in contact with patients starting 17 March;
- Isolation and cohorting of patients with COVID-19 in a dedicated area;
- Personal protective equipment for HCWs in charge of COVID-19 patients: gowns and gloves in addition to universal measures, a well-fitted respirator and eye protection during aerosol-generating procedures;
- Low-threshold screening by nasopharyngeal swab in HCWs for rhinitis, sore throat, cough, dyspnea, fever > 38 degrees or anosmia.

Daily assessment of patients for COVID-19 symptoms was performed for early detection of suspected cases. A low threshold was used to perform a nasopharyngeal swab for real-time reverse transcription polymerase chain reaction (rRT-PCR) analysis.

### 2.3. Population

A retrospective case series analysis of all the patients who tested positive for SARS-CoV-2 (rRT-PCR) between 15 March 2020, and 23 April 2020, was performed. All the cases were considered to be nosocomial (i.e., infection acquired at least 5 days or more prior to hospital admission). SARS-CoV-2 infections in HCWs that were diagnosed during this period were recorded simultaneously.

### 2.4. Diagnosis of SARS-CoV-2 Infection

Diagnosis was made using nasopharyngeal swabs by skilled clinical staff (a nurse or a medical resident).

SARS-CoV-2 was detected using an automated molecular diagnostic platform targeting the E gene or with the cobas SARS-CoV-2 test using a cobas 6800 instrument (Roche, Basel, Switzerland). The viral load was quantified based on cycle threshold values (Cts) and expressed in log^10^ following the equation derived from RNA quantification: −0.27 Ct + 13.04 [16]. For instance, a value of 1000 copies/mL corresponds to Cts > 37.

### 2.5. Symptoms Identification

Skilled clinical staff performed daily assessment of potential typical and atypical symptoms in all the patients. The symptoms present over the 7 days preceding and 14 days following a positive test were retrieved from the electronic medical records by a single investigator.

The symptomatic patients were defined as having typical COVID-19-related symptoms if presenting with any of a fever over 37.8 °C (100.0 °F), cough or dyspnea in the preceding seven days. The symptomatic patients were defined as having atypical symptoms if presenting with any of increased confusion, rhinorrhea, anosmia, dizziness, malaise, headache, nausea or diarrhea in the preceding week.

The patients were further classified as fully asymptomatic if they did not have any typical or atypical COVID-19-related symptoms at the time of testing and thereafter. The patients were classified as presymptomatic if they were asymptomatic at the time of testing but developed symptoms within two weeks after testing.

All the analyses were completed with the STATA software, version 16. The patients or their proxies (in cases of inability to provide consent, *n* = 2) provided written consent. The study was approved by the Cantonal Commission on Ethics in Human Research (IP 2020–02040).

## 3. Results

Between 15 March 2020, and 23 April 2020, a total of 152 screening smears were performed, leading to the identification of 34 patients infected with SARS-CoV-2, all nosocomial cases.

**Figure 1:**
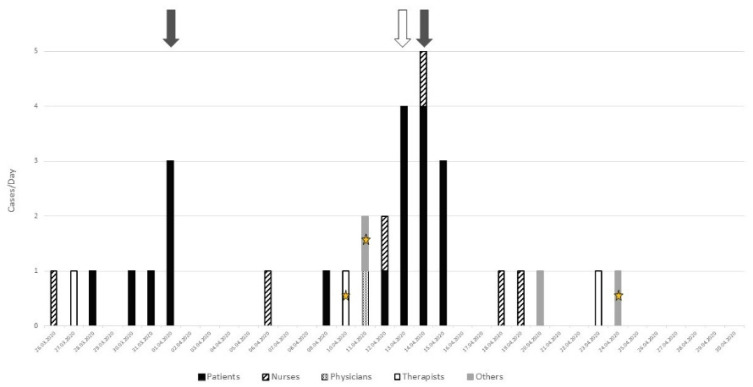
Analysis of the two clusters on the 6th floor: grey arrow = systematic screening, white arrow = contact screening (i.e., patients who have been in contact with sick caregivers), star = three health professionals working on two different floors.

On March 26, the first HCW working on the 6th floor tested positive for SARS-CoV-2 (Figure 1). The next day, another HCW tested positive. Over the next five days, the first cluster of six patients was detected, including three detected by systematic screening (grey arrow) triggered by the clustering of the first cases in the patients and the professionals. One week later, the second cluster of thirteen patients was identified on the same floor. Four cases were detected by contact screening (white arrow) and four—by systematic screening. Over this period, eleven additional HCWs were infected, including physicians, nurses, physical and occupational therapists, as well as other professionals. Overall, 19 patients and 13 professionals were infected during this cluster on the 6th floor.

**Figure 2:**
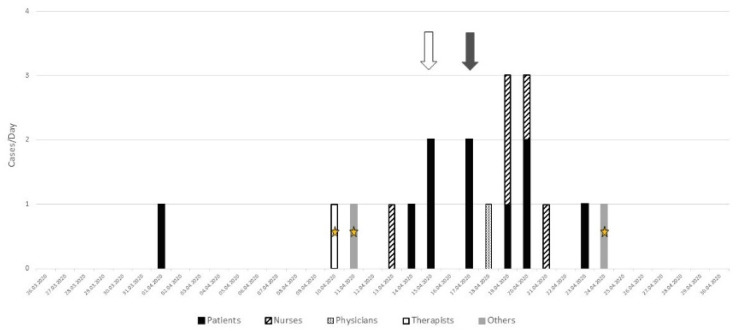
Analysis of the cluster on the 8th floor: grey arrow = systematic screening, white arrow = contact screening (i.e., patients who have been in contact with sick caregivers), star = three health professionals working on two different floors.

On April 10, another cluster started on the 8th floor (Figure 2) with two HCWs who also provided care on the 6th floor. Over the following days, a cluster of nine patients was detected, two of them by systematic screening (grey arrow) and two—by contact screening (white arrow). The other five patients were detected because of symptoms. Seven additional professionals infected with SARS-CoV-2 were also identified, among whom one also provided some care on the 6th floor.

In total, twenty-eight infections among the patients occurred in three separate outbreaks, and the remaining six were isolated cases. Over the same period, 19 professionals (including physicians, nurses, physical and occupational therapists, dieticians, and hospital cleaners) were diagnosed with COVID-19. All were symptomatic and had a simple disease course (i.e., without the need to be hospitalized).

Consent was obtained from 27 patients to further investigate their clinical characteristics (Table 1). The median age was 87 years (range, 66–98), all were Caucasian, and two thirds (18/27) were women. All the patients lived at home before the admission and most were impaired in mobility and in performing their daily functions. Most patients had at least one comorbidity, most frequently hypertension or cardiovascular disease (cardiac, cerebral or peripheral artery disease), but only two were obese.

At the time of diagnosis, seven out of the 27 patients (26%) were asymptomatic, but only one remained fully asymptomatic over the next days. Among the six presymptomatic patients, the median time between positive RT-PCR and symptoms was 2 days (range, 0.5–5). Among the 20 patients who were symptomatic, most (17/20) had typical symptoms, and only 3/20 had atypical symptoms.

**Figure 3:**
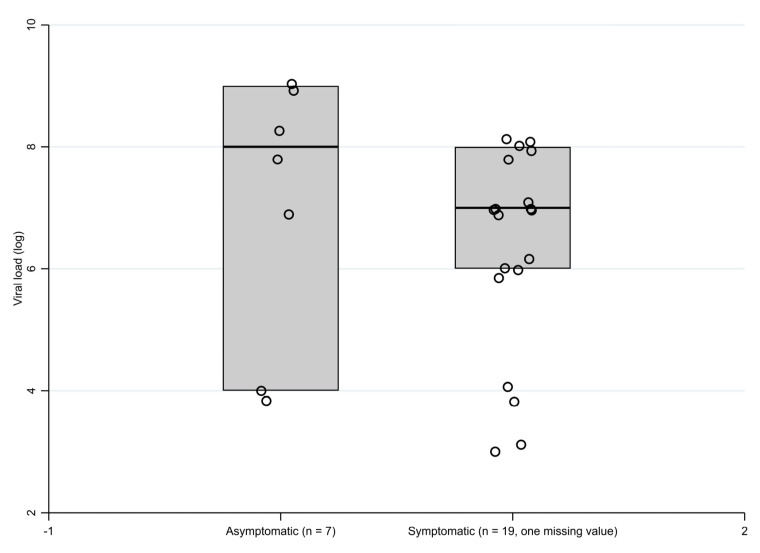
Boxplot of the viral load in asymptomatic and symptomatic patients.

The proportion of patients with the viral load >10^6^ copies/mL did not differ in patients with and without symptoms (15/19 vs. 5/7, one value missing), see Figure 3. Interestingly, one third of the infected patients had a very high viral load (≥10^8^ copies/mL).

Regarding the eight patients who died, all did so within 30 days of COVID-19 diagnosis (median, 10 days; range, 1–25). Seven died from respiratory failure, and one—from a probable stroke. The CT value/estimated viral load for these patients ranged from 10^3^ to 10^8^ copies/mL.

## 4. Discussion

The analysis of this outbreak of SARS-CoV-2 at a geriatric rehabilitation facility strongly suggests that bidirectional transmission likely occurred between HCWs and patients despite the implementation of protective measures. These results in a rehabilitation setting extend previous observations of outbreaks in skilled nursing and long-term facilities that also reported rapid transmission of SARS-CoV-2 among their vulnerable population [3,25]. Indeed, patients at rehabilitation facilities appear at particularly increased risk of nosocomial transmission because of their health, mobility and functional profile that requires close and prolonged care, as well as personal assistance. Importantly, at the time of these outbreaks, the hospital policy did not require that patients wear a mask during care and rehabilitation sessions, a measure that was implemented later during the pandemic. Moreover, no systematic eye protection was mandated for HCWs when providing close care [26].

Another contribution of this study is that it highlights the significant proportion (26%) of older patients who were initially asymptomatic with a high viral load and therefore already contagious. These results further underscore the increased risk of nosocomial transmission of SARS-CoV-2 in similar older populations [11,12]. Indeed, the proportion of initially asymptomatic patients is very similar to that found in other studies in older persons [3,13]. The median time to symptom onset appeared shorter than in other studies (2 days here vs. about 5 days) but is highly dependent on the timing of repeated and systematic screening [4].

Finally, the results also extend findings from other studies [3,8,14] that reported little or no difference in the viral load in a- or presymptomatic vs. symptomatic patients at the time of testing. In our population, about three quarters of the patients had a viral load > 10^6^ copies/mL regardless of the group to which they belonged (a-/presymptomatic versus symptomatic). Although several studies suggest that the viral load has no impact on the disease’s evolution and severity [27] and that infectivity during the course of the disease does not necessarily correlate with viral shedding [28], it remains possible that a high initial viral load is associated with higher contagiousness [29].

This study has several limitations such as its retrospective design and small sample size. However, this small sample size may also reflect the effectiveness of the measures implemented (systematic screening and screening at the slightest symptoms). A further limitation is the lack of concurrent systematic screening of asymptomatic HCWs at the time of these initial outbreaks, a measure that was implemented later [30]. This shortcoming makes it impossible to describe in more detail the precise role of HCWs in this outbreak, even though the chronology of the cases outbursts on both floor leaves little doubt about this role. Furthermore, Ct values provide an accurate estimate of the viral load but are not as precise as a quantitative test using a standard curve.

This study also has some strengths such as the updated list of symptoms that was used during the outbreaks that ensured precise collection of typical as well as atypical symptoms. A single investigator collecting data from medical records ensured the absence of interobserver variations.

## 5. Conclusions

This work sheds light on the nosocomial transmission of SARS-CoV-2 and fills the gap in the information regarding the geriatric rehabilitation setting. The results show that patients hospitalized in this setting are at increased risk for nosocomial SARS-CoV-2 transmission and support a low-threshold policy to systematically screen patients as soon as the first nosocomial case occurs. Early systematic screening in HCWs and mask wearing by patients needs to be studied further. Increasing vaccination against COVID-19 among patients and HCWs will further help to limit the risk of transmission within rehabilitation facilities.

## Figures and Tables

**Table 1:** Characteristics of the patients.

Characteristics	Total
	*n* = 27
Age	
Mean (±SD)	85.6 (±9.3)
Median (min–max)	87.3 (66.1–98.1)
Sex, *n* (%)	
Women	18 (66.7)
Men	9 (33.3)
Geriatric syndromes, *n* (%)	
Cognitive impairment *	15 (57.7)
Depressive symptoms ^†^	7 (26.9)
Mobility impairment ^‡^	26 (96.3)
Impaired nutritional status ^§^	14 (58.3)
Functional impairment in basic ADLs ^‖^	17 (63.0)
Functional impairment in instrumental ADL ^Ⅱ^	24 (88.9)
Comorbidities, *n* (%)	
Hypertension	16 (59.3)
Cardiovascular disease	15 (55.6)
Chronic respiratory disease	5 (18.5)
Immunodeficiency	4 (14.8)
Obesity	2 (7.4)
Diabetes	4 (14.8)
None	4 (14.8)
Discharge destination	
Died	8 (29.6)
Nursing home	8 (29.6)
Home	11 (40.7)

* Defined as the Mini-Mental State Examination score (MMSE) < 24 [17], the Montreal Cognitive Assessment score (MOCA) < 26 [18] or the MiniCog score < 3 [19]. ^†^ Defined as a score > 0 on the mini Geriatric Depression Scale (GDS) [20]. ^‡^ Defined as a score ≤ 23 according to Tinetti’s performance-oriented mobility assessment (POMA) [21]. ^§^ Defined as the Mini Nutritional Assessment score ≤ 7 [22]. ^‖^ Defined as impairment in at least one out of six basic activities of daily living (ADL) [23]; include dressing, toileting, going to the WC, transferring, maintaining continence, eating; score range—from 0 to 6, with higher scores indicating better function. ^Ⅱ^ Defined as impairment in at least one out of eight instrumental activities of daily living (ADL) [24]; include using the phone, managing finances, managing medications, preparing meals, doing the laundry, cleaning, shopping and using the transportation; score range—from 0 to 8, with higher scores indicating better function.

## Data Availability

The datasets analyzed during this study are available from the corresponding author upon reasonable request.

## References

[1] Meyerowitz E.A., Richterman A., Gandhi R.T., Sax P.E. (2021). Transmission of SARS-CoV-2: A Review of Viral, Host, and Envi-ron-mental Factors. Ann. Intern. Med..

[2] Tang S., Mao Y., Jones R.M., Tan Q., Ji J., Li N., Shen J., Lv Y., Pan L., Ding P. (2020). Aerosol transmission of SARS-CoV-2? Evidence, prevention and control. Environ. Int..

[3] Arons M.M., Hatfield K.M., Reddy S.C., Kimball A., James A., Jacobs J.R., Taylor J., Spicer K., Bardossy A.C., Oakley L.P. (2020). Presymptomatic SARS-CoV-2 Infections and Transmission in a Skilled Nursing Facility. N. Engl. J. Med..

[4] Lauer S.A., Grantz K.H., Bi Q., Jones F.K., Zheng QMeredith H.R., Azman A.S., Reich N.G., Lessler J. (2020). The incubation period of coronavirus disease 2019 (COVID-19) from publicly reported confirmed cases: Estimation and application. Ann. Intern. Med..

[5] He X., Lau E.H.Y., Wu P., Deng X., Wang J., Hao X., Lau Y.C., Wong J.Y., Guan Y., Tan X. (2020). Temporal dynamics in viral shedding and transmissibility of COVID-19. Nat. Med..

[6] Annweiler C., Sacco G., Salles N., Aquino J.-P., Gautier J., Berrut G., Guérin O., Gavazzi G. (2020). National French Survey of Coronavirus Disease (COVID-19) Symptoms in People Aged 70 and Over. Clin. Infect. Dis..

[7] Martín-Sánchez F.J., Del Toro E., Cardassay E., Carbó A.V., Cuesta F., Vigara M., Gil P., Picado A.L.L., Valero C.M., Miranda J.D. (2020). Clinical presentation and outcome across age categories among patients with COVID-19 admitted to a Spanish Emergency Department. Eur. Geriatr. Med..

[8] Lavezzo E., Franchin E., Ciavarella C. (2020). Suppression of a SARS-CoV-2 outbreak in the Italian municipality of Vo’. Nature.

[9] Oran D.P., Topol E.J. (2020). Prevalence of Asymptomatic SARS-CoV-2 Infection: A Narrative Review. Ann. Intern Med..

[10] Koh W.C., Naing L., Chaw L., Rosledzana M.A., Alikhan M.F., Jamaludin S.A., Amin F., Omar A., Shazli A., Griffith M. (2020). What do we know about SARS-CoV-2 transmission? A systematic review and meta-analysis of the secondary attack rate and associated risk factors. PLoS ONE.

[11] Harris B.H.L., Zuhair M., Di Giovannantonio M., Rosadas C., Khan M., Short C.-E., Thaventhiran T., Quinlan R., Taylor A., Calvez R. (2020). Asymptomatic COVID-19 in a rehabilitation facility: Evolution of the presence of nasopharyngeal SARS-CoV-2 and serological antibody responses. J. Infect. Dis..

[12] Kirshblum S.C., Mph G.D., Lopreiato M.C., Pomeranz B., Dawson A., Hammerman S., Gans B.M. (2020). Screening Testing for SARS-CoV-2 upon Admission to Rehabilitation Hospitals in a High COVID-19 Prevalence Community. PM&R.

[13] Kimball A., Hatfield K.M., Arons M. (2020). Asymptomatic and presymptomatic SARS-CoV-2 infections in residents of a long-term care skilled nursing facility—King County, Washington, March 2020. Morb. Mortal. Wkly. Rep..

[14] Walsh K.A., Jordan K., Clyne B., Rohde D., Drummond L., Byrne P., Ahern S., Carty P.G., O’Brien K.K., O’Murchu E. (2020). SARS-CoV-2 detection, viral load and infectivity over the course of an infection. J. Infect..

[15] Zhou R., Li F., Chen F., Liu H., Zheng J., Lei C., Wu X. (2020). Viral dynamics in asymptomatic patients with COVID-19. Int. J. Infect. Dis..

[16] Jacot D., Greub G., Jaton K., Opota O. (2020). Viral load of SARS-CoV-2 across patients and compared to other respiratory viruses. Microbes Infect..

[17] Folstein M.F., Folstein S.E., McHugh P.R. (1975). “Mini-mental state”: A practical method for grading the cognitive state of patients for the clinician. J. Psychiatr. Res..

[18] Nasreddine Z.S., Phillips N.A., Bã©Dirian V., Charbonneau S., Whitehead V., Collin I., Cummings J.L., Chertkow H. (2005). The Montreal Cognitive Assessment, MoCA: A Brief Screening Tool For Mild Cognitive Impairment. J. Am. Geriatr. Soc..

[19] Borson S., Scanlan J., Brush M., Vitaliano P., Dokmak A. (2000). The mini-cog: A cognitive ‘vital signs’ measure for dementia screening in multi-lingual elderly. Int. J. Geriatr. Psychiatry.

[20] Clément J.P., Nassif R.F., Léger J.M., Marchan F. (1997). Mise au point et contribution à la validation d’une version française brève de la Geriatric Depression Scale de Yesavage [Development and contribution to the validation of a brief French version of the Ye-savage Geriatric Depression Scale]. Encephale.

[21] Tinetti M.E. (1986). Performance-Oriented Assessment of Mobility Problems in Elderly Patients. J. Am. Geriatr. Soc..

[22] Rubenstein L.Z., Harker J.O., Salvà A., Guigoz Y., Vellas B. (2001). Screening for Undernutrition in Geriatric Practice: Developing the Short-Form Mini-Nutritional Assessment (MNA-SF). J. Gerontol. Ser. A Boil. Sci. Med. Sci..

[23] Katz S. (1983). Assessing Self-maintenance: Activities of Daily Living, Mobility, and Instrumental Activities of Daily Living. J. Am. Geriatr. Soc..

[24] Lawton M.P., Brody E.M. (1969). Assessment of Older People: Self-Maintaining and Instrumental Activities of Daily Living. Gerontologist.

[25] McMichael T.M., Currie D.W., Clark S., Pogosjans S., Kay M., Schwartz N.G., Lewis J., Baer A., Kawakami V., Lukoff M.D. (2021). Epidemiology of COVID-19 in a Long-Term Care Facility in King County, Washington. N. Engl. J. Med..

[26] European Centre for Disease Prevention and Control (2021). Infection Prevention and Control and Preparedness for COVID-19 in Healthcare Settings—Sixth Update.

[27] Argyropoulos K.V., Serrano A., Hu J., Black M., Feng X., Shen G., Call M., Kim M.J., Lytle A., Belovarac B. (2020). Association of Initial Viral Load in Severe Acute Respiratory Syndrome Coronavirus 2 (SARS-CoV-2) Patients with Outcome and Symptoms. Am. J. Pathol..

[28] Widders A., Broom A., Broom J. (2020). SARS-CoV-2: The viral shedding vs infectivity dilemma. Infect. Dis. Health.

[29] Avadhanula V., Nicholson E.G., Ferlic-Stark L., Piedra F.A., Blunck B.N., Fragoso S., Bond N.L., Santarcangelo P.L., Ye X., McBride T.J. (2021). Viral load of SARS-CoV-2 in adults during the first and second wave of COVID-19 pandemic in Houston, TX: The potential of the super-spreader. J. Infect. Dis..

[30] Swissnoso Management and Control of COVID-19 Outbreaks in Healthcare Settings. https://www.swissnoso.ch/fileadmin/swissnoso/Dokumente/5_Forschung_und_Entwicklung/6_Aktuelle_Erreignisse/210329_Control_of_healthcare-associated_COVID-19_outbreaks_V2.0.pdf.

